# Immune Response After Cochlear Implantation

**DOI:** 10.3389/fneur.2020.00341

**Published:** 2020-05-14

**Authors:** Edi Simoni, Erica Gentilin, Mariarita Candito, Giulia Borile, Filippo Romanato, Milvia Chicca, Sara Nordio, Marta Aspidistria, Alessandro Martini, Diego Cazzador, Laura Astolfi

**Affiliations:** ^1^Bioacoustics Research Laboratory, Department of Neurosciences, University of Padua, Padua, Italy; ^2^Otorhinolaryngology Unit, Department of Neurosciences, University of Padua, Padua, Italy; ^3^Section of Human Anatomy, Department of Neuroscience, University of Padua, Padua, Italy; ^4^Department of Physics and Astronomy “G. Galilei”, University of Padua, Padua, Italy; ^5^Laboratory for Nanofabrication of Nanodevices, Padua, Italy; ^6^Department of Life Sciences and Biotechnology, University of Ferrara, Ferrara, Italy; ^7^Fondazione Ospedale San Camillo IRCCS, Venice, Italy; ^8^Department of Statistical Sciences, University of Padua, Padova, Italy

**Keywords:** drug delivery, fibrosis, cochleostomy, neuronal degeneration, steroids, dexamethasone

## Abstract

A cochlear implant (CI) is an electronic device that enables hearing recovery in patients with severe to profound hearing loss. Although CIs are a successful treatment for profound hearing impairment, their effectivity may be improved by reducing damages associated with insertion of electrodes in the cochlea, thus preserving residual hearing ability. Inner ear trauma leads to inflammatory reactions altering cochlear homeostasis and reducing post-operative audiological performances and electroacoustic stimulation. Strategies to preserve residual hearing ability led to the development of medicated devices to minimize CI-induced cochlear injury. Dexamethasone-eluting electrodes recently showed positive outcomes. In previous studies by our research group, intratympanic release of dexamethasone for 14 days was able to preserve residual hearing from CI insertion trauma in a Guinea pig model. Long-term effects of dexamethasone-eluting electrodes were therefore evaluated in the same animal model. Seven Guinea pigs were bilaterally implanted with medicated rods and four were implanted with non-eluting ones. Hearing threshold audiograms were acquired prior to implantation and up to 60 days by recording compound action potentials. For each sample, we examined the amount of bone and fibrous connective tissue grown within the scala tympani in the basal turn of the cochlea, the cochleostomy healing, the neuronal density, and the correlation between electrophysiological parameters and histological results. Detection of tumor necrosis factor alpha, interleukin-6, and foreign body giant cells showed that long-term electrode implantation was not associated with an ongoing inflammation. Growth of bone and fibrous connective tissue around rods induced by CI was reduced in the scala tympani by dexamethasone release. For cochleostomy sealing, dexamethasone-treated animals showed less bone tissue growth than negative. Dexamethasone did not affect cell density in the spiral ganglion. Overall, these results support the use of dexamethasone as anti-inflammatory additive for eluting electrodes able to protect the cochlea from CI insertion trauma.

## Introduction

The loss of cochlear hair cells invariably leads to sensorineural hearing loss because no niches of stem cells able to renew this tissue have been identified to date in the organ of Corti (1). Thus, the only way to restore hearing ability is to undergo a cochlear implant (CI) surgery, and currently, the application of this device is useful for young as well as old patients (2, 3). Unfortunately, this advanced electroacoustic device may cause adverse effects, among which damages due to insertion of the electrode into the cochlea. Such damages may be mechanical (disruption of basal membrane and spiral ligament) or physiological (residual hearing impairment, inflammatory foreign body reaction, and neuronal degeneration). In order to prevent or reduce these adverse effects, several innovative surgical approaches have been developed, such as different accesses for CI insertion (round window or cochleostomy) (4) and less traumatic (thinner and shorter) electrodes (5, 6). Anti-inflammatory drugs have been applied to prevent foreign body reaction (7–11) and to prevent neuronal degeneration (12). In a recent review on the complications of CI surgery, cochlear complications are defined as unusual, accounting for 1%. Only three cases of chronic granulating labyrinthitis and 19 cases of cochlear fibrosis/osteoneogenesis were found over 7,132 surgical procedures (13). Explantation of the array was necessary in all the abovementioned cases.

The inflammatory response to CI has been investigated in both animal models and humans, showing that it may cause tissue growth around the electrode (7, 11, 14, 15), inducing fluctuations or increase of the impedance level (16) or even electrode extrusion (13, 14, 17). The disruption of cochlear structures may also increase local inflammation, in turn leading to further tissue growth (16). The main cause for inflammatory reaction to the foreign body is thought to be silicone embedding the electrode (14). Previous studies by our research group showed that the inflammatory reaction was not due to toxicity of silicone or of its polydimethylsiloxane components, because these compounds were found biocompatible in an inner ear cell line derived from Immortomouse™ organ of Corti (OC-k3) and in a neuronal cell line derived from rat pheochromocytoma (PC12) (18, 19). Concerning cell adhesion, silicone-derived compounds have been shown able to support growth and surface cell adhesion in PC12 (20), but opposite results have also been reported (21).

The use of glucocorticoids is known to reduce cochlear damage and hearing loss caused by a traumatic lesion or by ototoxic drugs (22–25). Among drugs currently employed in auditory therapies, there is dexamethasone, recognized by glucocorticoid receptors, thus able to activate anti-inflammatory and anti-apoptotic pathways (26–30). Previous studies on a new dexamethasone-eluting electrode designed by MED-EL Hearing Implants (Innsbruck, Austria) showed an *in vitro* continuous release of the drug up to 60 days (7, 31) and *in vivo* in a Guinea pig model anti-inflammatory effects up to 14 days after cochleostomy (acute reaction) (7). Based on these results, we examined the *in vivo* long-term anti-inflammatory effects (chronic reaction) in the same animal model up to 60 days after cochleostomy.

## Materials and Methods

### Animals

Eleven tricolor Guinea pigs obtained from Charles River (Lecco, Italy) underwent cochleostomy and were implanted with medical grade silicone rods without any contact or wire. The animals were randomly divided into two experimental groups: the first one was implanted with 10% dexamethasone-eluting rods (DERs, 7 animals, 14 ears), and the second one was implanted with non-eluting rods (NERs, 4 animals, 8 ears).

All animal tests were approved according to Italian guidelines provided in DL 116/92, with reference to European Economic Community directive 86–609. The animals were treated by accepted veterinary standards and housed under the same living conditions.

### Drug Delivery Rods

The silicone rods were 25 mm long and 0.6 mm in diameter, with a 5-mm-long tip decreasing in diameter from 0.6 to 0.3 mm. In the DER group, the 5 mm tip of the silicone rod was composed of silicone mixed with 10% dexamethasone, while in the NER group, no drug was added to silicone as previously reported (7).

### Electrophysiological Measurements and Surgical Procedure

In order to verify the absence of hearing problems, the auditory threshold was measured before surgery by the auditory brainstem responses (ABRs) in all animals. The compound action potential (CAP) was used to assess the auditory threshold immediately before (pre-op) and after cochleostomy at days 0, 3, 7, 14, 30, and 60. Each animal was handled under strict aseptic conditions, and the CAP recording and the surgery approach were performed as previously described (6, 7).

Briefly, as anesthesia, 0.5 ml atropinsulfate (0.5 mg/ml) was intraperitoneally injected while 10 mg/kg enrofloxacin (5%) was subcutaneously administered. The cochleostomy was performed by retroauricolar approach, the hole diameter was 0.7 mm, and the rod was inserted at a depth of 3 mm. In order to record the CAP threshold, a gold wire was placed near the round window. To measure CAP thresholds, the stimuli were clicks and Gaussian-shaped tone pips presented in the frequency range of 0.5–32 kHz (2 points/octave) and at an intensity range from 10 to 100 dB (in 2-dB steps, 30 averages per step). During the recording procedure, the contralateral ear was blocked with a foam insert.

The CAP threshold evoked by click, low-frequency band (4- and 8-kHz tone pips) and high-frequency band (16- and 32-kHz tone pips), and the thresholds were measured immediately before (pre-op) and after cochleostomy at days 0, 3, 7, 14, 30, and 60. The threshold shifts (TSs) were calculated subtracting the pre-op CAP value from each CAP value measured after cochleastomy. A positive value of TS indicated a hearing loss while a negative one indicated a hearing recovery after cochleostomy.

The insertion trauma was evaluated by comparing CAP recordings between pre-op and day 0, and the possible damage or recovery was evaluated by comparing CAP recordings between pre-op and all the other time intervals.

### Histology

**Figure 1:**
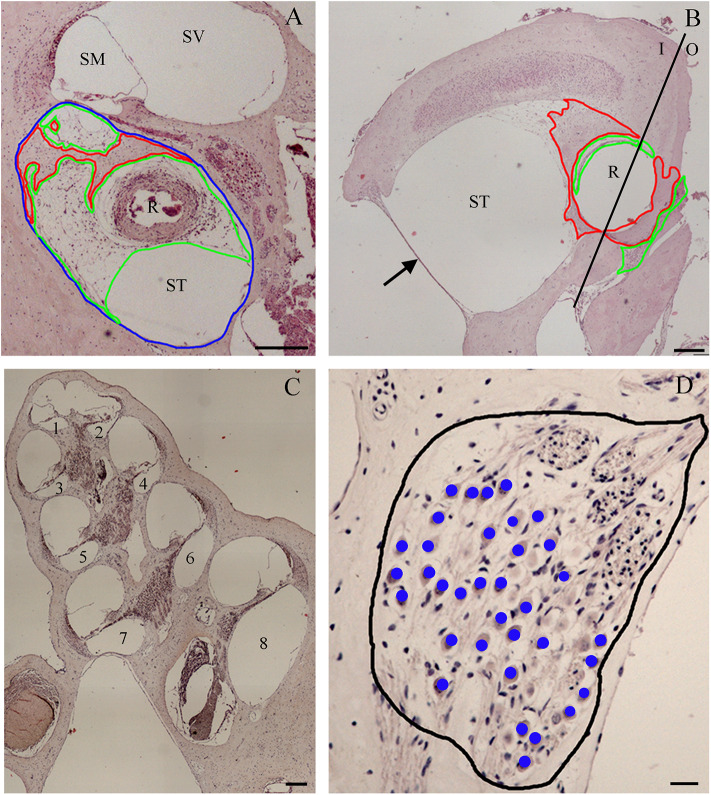
Tissue growth within the scala tympani **(A)** and around cochleostomy **(B)**. Section of the cochlear duct suitable to measure the neuron density in the spiral ganglion **(C)** and spiral ganglion neuron density **(D)**. Blue outline, scala tympani area; red outline, bone area; green outline, fibrotic area; R rod site; ST, scala tympani space; SV, scala vestibuli; SM, scala media; I, inside; O, outside; 1–4, apical regions; 5–6, medial regions; 7–8, basal regions; black outline, ganglion area; blue dots, neurons. Scale bars, 200 μm **(A–C)** and 20 μm **(D)**. Arrow, round window.

All animals were painlessly sacrificed by decapitation after the last CAP recording, when they were still anesthetized. The removed bullae were placed in Shandon Glyo-Fixx™ (Thermo Scientific, Milan, Italy) and incubated at 4°C up to 20 h. After fixation, the samples were decalcified by an EDTA solution (10% ethylene diamine tetra acetic acid in phosphate buffer, pH 7.4) for 28 days in an incubator at 37°C. The EDTA solution was changed every 2 days. After decalcification, the bullae were washed, dehydrated, and embedded in paraffin (Diapath S.p.A, Bergamo, Italy). Once oriented with the modiolus axis parallel to the longitudinal axis of the paraffin block, the samples were cut by a semiautomatic microtome CUT 5062 (SLEE medical GmbH, Mainz, Germany) in 5-μm sections that were sequentially collected on Superfrost ® Plus microscope slides (Diapath S.p.A), and each section was spaced 50 μm from the next one, to analyze the entire cochlea. For each cochlea, about 60 sections were stained with hematoxylin–eosin (EE). The Nis-Elements 3.0 Image Analysis System software (Nikon, Amsterdam, Netherlands) was employed for histological and biometrical analyses. The parameters analyzed for each cochlea section stained with EE were the following:

A. The amount of tissue growth within the scala tympani in the basal turn of the cochlea (TG, “occlusion”), expressed in μm^2^ and percentage (tissue area/scala tympani area). Both the fibrous and bone components of the tissue were included in the tissue growth (Figure 1A).
B. The amount of tissue growth around the cochleostomy hole (“healing”) expressed in μm^2^ (Figure 1B). For each section, the cochleostomy hole was divided in an internal and an external region (Figure 1B). Measurements were performed in all sections in which the cochleostomy hole was visible.
C. The neuronal density, expressed as the number of neurons/10,000 μm^2^. In order to measure the number of neurons, slices including the modiolus (the conic central axis of the cochlea) were divided into three regions, apical, medial, and basal, respectively, with four (1–4), two (5–6), and two (7–8) sections of the membranous labyrinth (Figure 1C). The neuronal density was calculated on three consecutive slices containing the modiolus: only neurons with nuclei were counted (Figure 1D). The number of neurons was divided by the area of the Rosenthal's canal.

The Nis-Elements 3.0 Image Analysis System software (Nikon) converts the pixels in micrometers based on the objective magnification and then calculates the size of a given distance or area. Based on these data, the amount of tissue growth within the scala tympani (expressed in area) was measured at the basal turn of the cochlea and correlated to the distance from the cochleostomy hole. The size of the cochleostomy hole and the thickness and the area of tissue growth around it were also measured.

Masson's trichrome staining (Bio-Optica, Milan, Italy) was applied to highlight the fibrotic reaction products in the scala tympani and around the cochleostomy hole (two samples: about 60 sections each, per group).

The label-free second harmonic generation analyses (detected by the two-photon microscope optimized as previously reported by Filippi and collaborators) (32) were applied to highlight the collagen and elastin production around the cochleostomy hole (two samples: about 10 sections each, per group).

### Immunohistochemistry

The sections were permeabilized with a 0.1% Tween 20 (Sigma-Aldrich, Milan, Italy) solution in PBS, endogenous peroxidase was blocked with 1% hydrogen peroxide (Marco Viti Pharmaceutical, Sandrigo, Vicenza, Italy), and non-specific sites were saturated with the blocking buffer Vecstain Elite ABC kit (Vector Laboratories, Burlingame, CA, USA). Sections were then incubated with the primary antibody at the concentrations 1:250 for IL-6 (bs-0782R, Bioss Inc, Woburn, Massachusetts, USA) and 1:200 for TNFalfa (ab1793, abcam, San Francisco, USA) overnight at 4°C.

After 24 h, the incubation with the appropriate secondary antibody Vecstain Elite ABC kit (Vector Laboratories) was performed, followed by signal amplification and detection with Vector SG Peroxidase substrate kit (Vector) and the Nuclear Fast Red (Vector Laboratories). Finally, after dehydration and a passage in xylene, the slides were mounted with Surgipath® Micromount (Leica Biosystem, Buccinasco, Milan, Italy) and observed with the optical microscope ECLIPSE 50i (Nikon). Two samples per groups were analyzed at the cochleostomy and mid-modiolar region (about 15 sections each). The images were acquired with Nis Elements D 3.2 software (Nikon).

### Statistical Analyses

Concerning the CAP TSs, the statistical significance among time intervals and experimental groups was measured by the Mann–Whitney *U* test (significant with *p* ≤ 0.05, highly significant with *p* ≤ 0.01).

The correlations between tissue growth (as scala tympani occlusion or cochleostomy healing) and hearing ability (as insertion trauma or TS recorded at day 60) were measured by Spearman non-parametric rank correlation. Statistical significance between experimental groups was measured by the Kruskal–Wallis one-way analysis assessing the differences between multiple groups. Correlations were established among the hearing TSs (expressed in dB and measured both at the click and at 32 kHz), the tissue growth (measured as both occlusion and healing), and the neuronal density at the insertion site. Linear regression models were applied to identify the influence of the tested variables on the TS at day 60. Statistical analyses were performed by the STATISTICA 7.1 software (Stat Soft Italia srl, Padua, Italy) and R software version 3.2.5 (33).

## Results

### Electrophysiology

**Figure 2:**
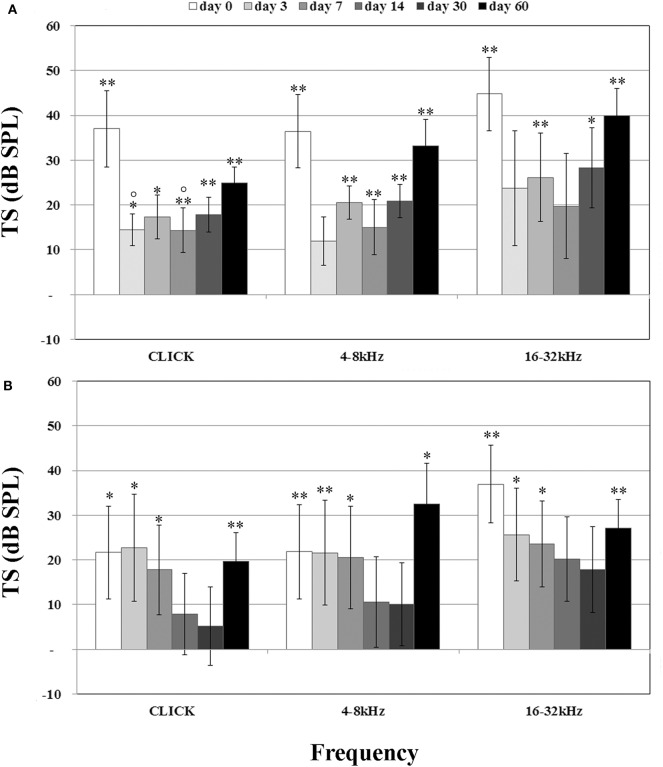
Compound action potential (CAP) related to treatment, reported as threshold shift (TS) and evaluated in comparison with CAP before cochleostomy (pre-op). **(A)** Negative eluting rod (NER) group. **(B)** Dexamethasone-eluting rod (DER) group. Significant differences referred to pre-op CAP measured before cochleostomy (insertion trauma): *p < 0.05, **p < 0.01. Significant differences referred to day 0 after cochleostomy (recovery): °*p* < 0.05. Values are shown as averages with standard error bars.

Before treatments, all Guinea pigs had normal hearing thresholds. When the animals were assigned to the experimental groups, no significant difference in hearing thresholds was detected among groups (*p* > 0.05). In the NER group, the significant TSs observed at day 0 were higher than 35 dB at all frequencies tested (Figure 2A). Over time, a quick recovery of TSs was observed after cochleostomy but also a significant increase 60 days post-surgery, with values similar to those detected at day 0. In the DER group immediately after cochleostomy, significant TSs (day 0) were observed at the high frequencies of about 35 dB, and at the click and lower frequencies of about 20 dB of TSs (Figure 2B). Over time, at all frequencies tested, the TSs decreased up to day 30 and then increased up to 60 days post-surgery (Figure 2B). Comparing the two treatment groups, although not statistically significant, at all frequencies tested after cochleostomy and over time, the DER group showed lower TSs than the NER one (Figure 2).

### Tissue Growth in the Scala Tympani

**Figure 3:**
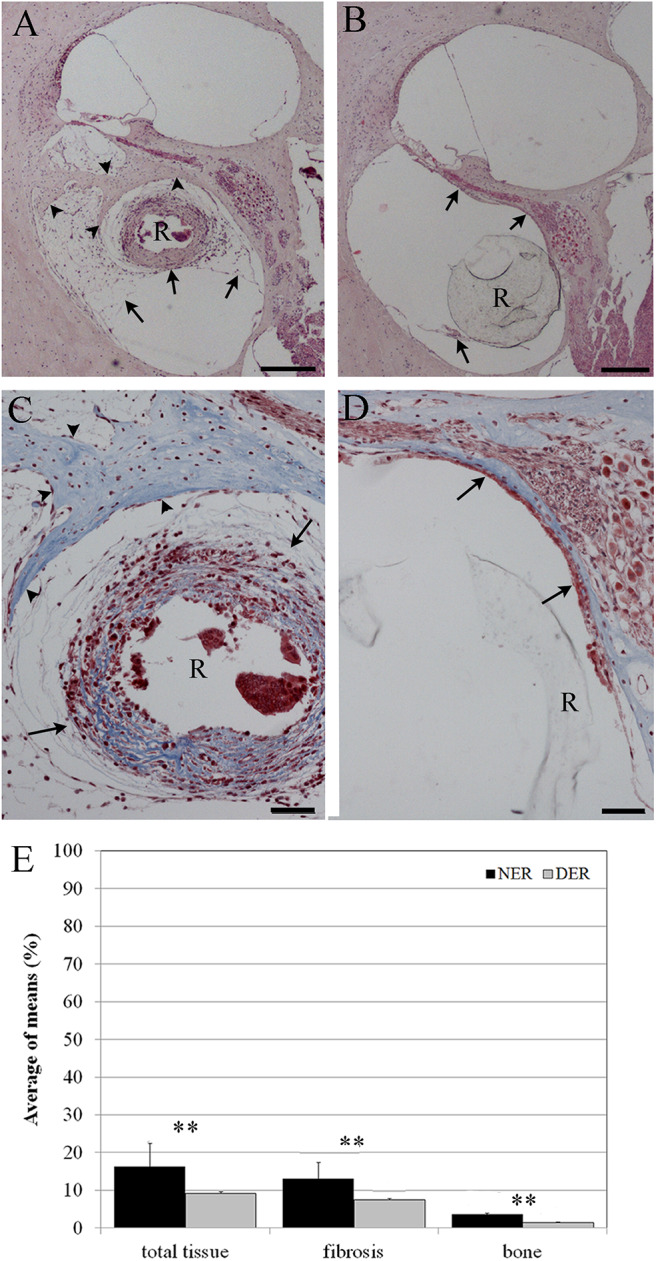
Tissue growth in the scala tympani according to treatment after 60 days. **(A,C)** NER group. **(B,D)** DER group. **(E)** Bar graph represents the scala tympani occlusion expressed as percentage of tissue growth in the scala tympani 60 days after surgery, according to treatment. Significant differences **p < 0.01. **(A,B)** Hematoxylin–eosin staining; scale bars, 200 μm. **(C,D)** Masson's trichrome staining; scale bars, 50 μm. Arrowheads, new bone growth; arrows, fibrotic tissue; R, rod site or silicon rod.

In order to evaluate the occlusion due to the formation of new tissue, we measured the amount of tissue growth as percentage of the scala tympani area. In the scala tympani of the NER group, a strong inflammatory reaction was observed around the electrode occluding almost all the scala tympani (Figure 3A). A high number of fibrotic cells and extracellular matrix deposition were observed nearby the rod site (Figure 3C), and outwardly a growth of new tissue bone was observed until the edge of scala tympani (Figures 3A,C). In the DER group, the inflammatory reaction was markedly reduced and characterized by fibrotic tissue mostly composed of fibroblasts tight to the edge of scala tympani (Figure 3D). In the same group, the average tissue growth measured in the scala tympani was significantly lower in comparison to NER; moreover, a significant reduction was observed in fibrotic tissue growth (fibrosis) and new bone formation (Figure 3E). In the DER group, the rod was still present, while in the NER group, it seemed to be replaced with fibrotic tissue (Figures 3A,B).

**Figure 4:**
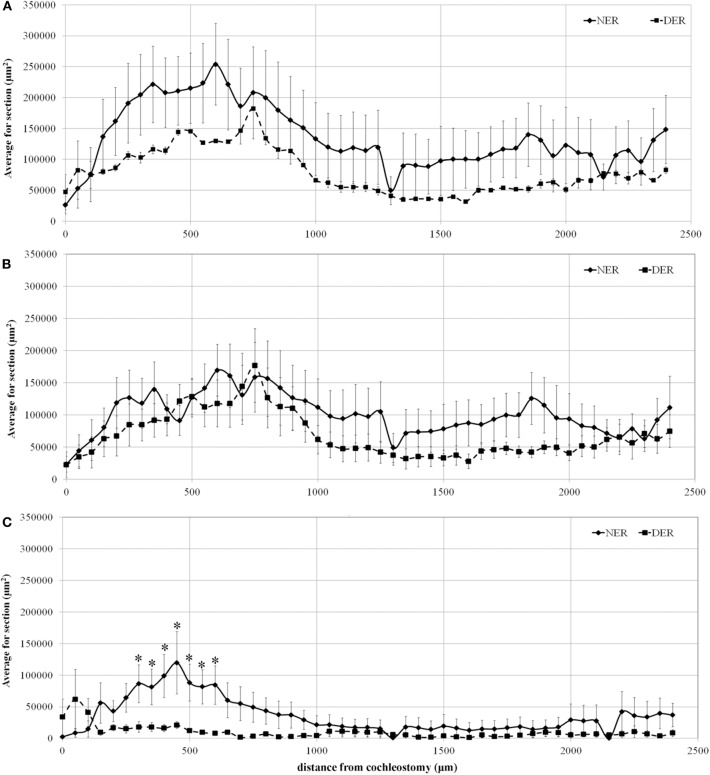
Tissue growth in the scala tympani along the basal turn of the cochlea. **(A)** Total tissue growth; **(B)** fibrotic tissue growth; **(C)** new bone formation. Significant differences between NER and DER groups: *p < 0.05. Values are shown as averages with standard error bars.

Concerning the correlation between the percentage of TG and its distance from the cochleostomy, the total TG, the fibrosis, and the area of new bone formation (expressed in μm^2^) are shown in Figure 4, plotted according to their distance from the cochleostomy site.

The data show that the areas of total TG progressively increased from the site of cochleostomy, peaking at about 600 μm. Tissue growth area until the end of the basal turn was always higher in NER than in DER, although not significant (Figure 4A). A similar amount of fibrotic tissue growth was observed near the cochleostomy, increasing in the NER group until the end of the basal turn at the cochlea, without reaching statistical significance (Figure 4B). The formation of new bone was significantly higher in NER in comparison to DER near the cochleostomy site, although there was a high variability within the groups. At a distance higher than 1,000 μm from the cochleostomy hole, the amount of new bone formation was similar between the groups (Figure 4C).

### Effects of Dexamethasone Elution on Cochleostomy Healing

**Figure 5:**
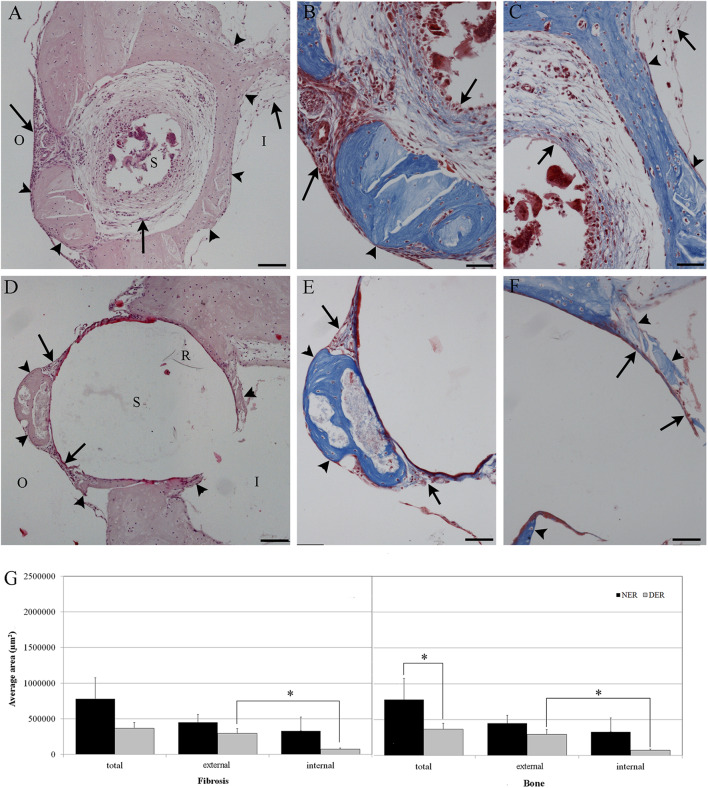
Cochleostomy sealing measured as tissue growth around the cochleostomy site after 60 days, according to treatment. **(A,D)** HEmatosillin-Eosin staining, **(B,C,E,F)** Masson's trichrome staining. **(A–C)** NER group. **(D–F)** DER group. **(G)** Bar graph represents the cochleostomy sealing expressed as areas (μm^2^) of fibrotic tissue and new bone growth around the cochleostomy site 60 days after surgery, according to treatment. Significant differences *p < 0.05. **(A,D)** Hematoxylin-eosin staining; scale bars, 100 μm. **(B,C,E,F)** Masson's trichrome staining; scale bars, 50 μm. I, inside cochleostomy; O, outside cochleostomy; S, rod site; R, silicon rod. Arrowheads, new bone growth; arrows, fibrotic tissue.

After 60 days from surgery, new bone formation mixed with fibrotic tissue around the cochleostomy site was detected in all samples. The EE and Masson's trichrome staining showed a higher inflammatory reaction in NER (Figures 5A–C) in comparison to DER (Figures 5D–F). The cochleostomy sealing was also measured for each cochlea as the amount of tissue growth, fibrosis, and new bone formation in the area inside and outside of the cochleostomy (Figure 1B). In the DER group, the amount of total new bone formation was significantly lower than in NER. Moreover, DER showed significantly lower amounts of fibrotic tissue and new bone formation inside the cochleostomy area than in the outside (*p* < 0.01), while in the NER group, the amount of these tissues was similar in both inside and outside areas (Figure 5G).

**Figure 6:**
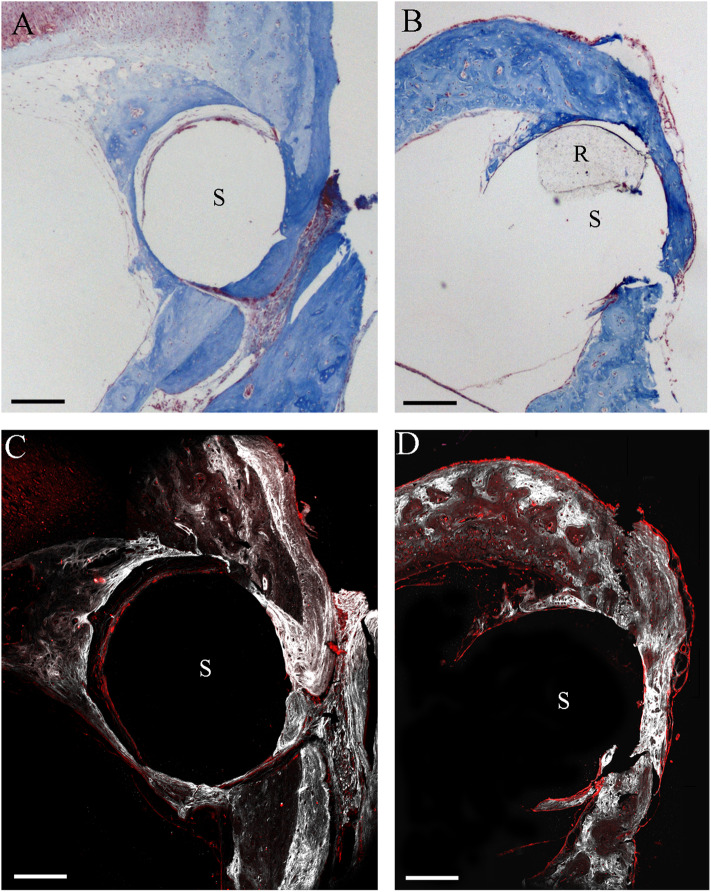
Two-photon microscope analysis. Masson's trichrome staining in NER **(A)** and DER **(B)** groups, respectively. Images obtained through label-free second harmonic generation captured at the same cochleostomy region, respectively, in a NER **(C)** and a DER **(D)** implant. In white, signal from collagen; in red, elastin (autofluorescence). S, rod site; R, silicon rod. Scale bars, 200 μm.

By the two-photon microscope analysis, it was possible to detect a larger second harmonic generation signal proportional to a higher deposition of collagen in the NER group than in the DER one around cochleostomy. Starting from the rod site, elastin and, outwardly, collagen were observed (Figure 6).

### Spiral Ganglion Integrity

**Figure 7:**
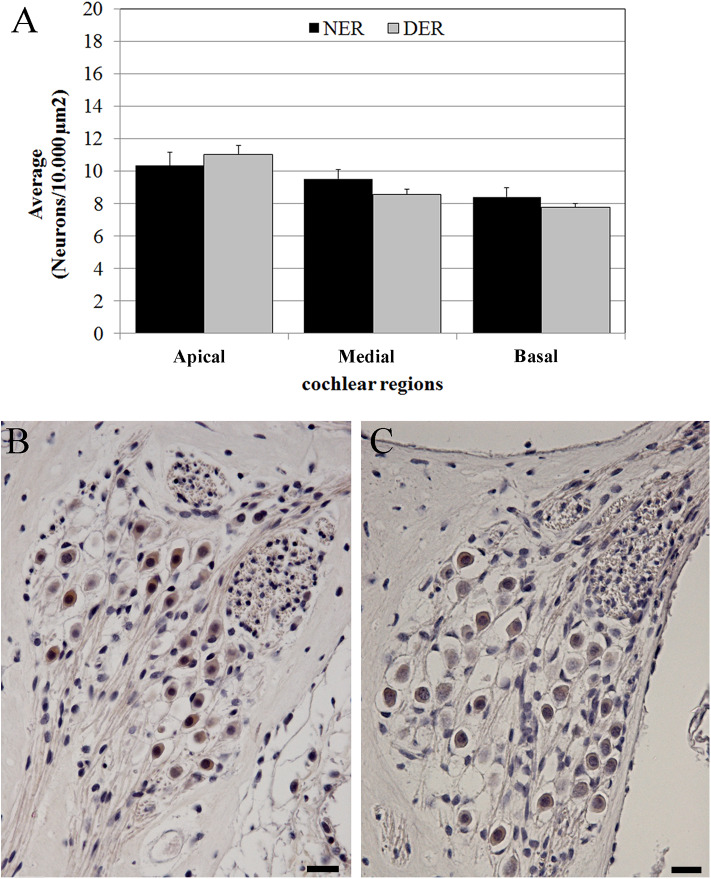
Neuronal density. **(A)** Average neuronal density measured in three regions of the cochlear turns. Bars represent standard errors. **(B)** NER, spiral ganglion in the basal turn. **(C)** DER, spiral ganglion in the basal turn. Scale bars, 20 μm.

The neuronal density was measured according to the cochlear region (basal, medial and apical turn) (Figures 1C,D) and no significant differences were detected between NER and DER in any region (*p* > 0.05) (Figure 7).

### Immunoreaction

**Figure 8:**
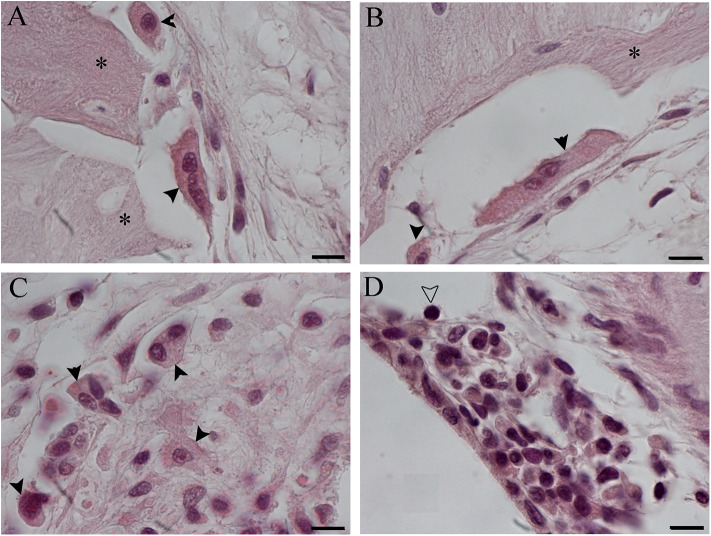
Foreign body giant cells (FBGCs) in proximity of new bone growth in the outer **(A)** and inner **(B)** side of the cochelostomy. Numerous FBGCs are visible also inside the inflammatory reaction around the electrode **(C)**. An inflammatory infiltration is visible near the cochleostomy **(D)**. Hematoxylin–eosin staining. Scale bars, 10 μm. *, new bone; black arrowheads, FGBC; white arrowhead, lymphocyte.

**Figure 9:**
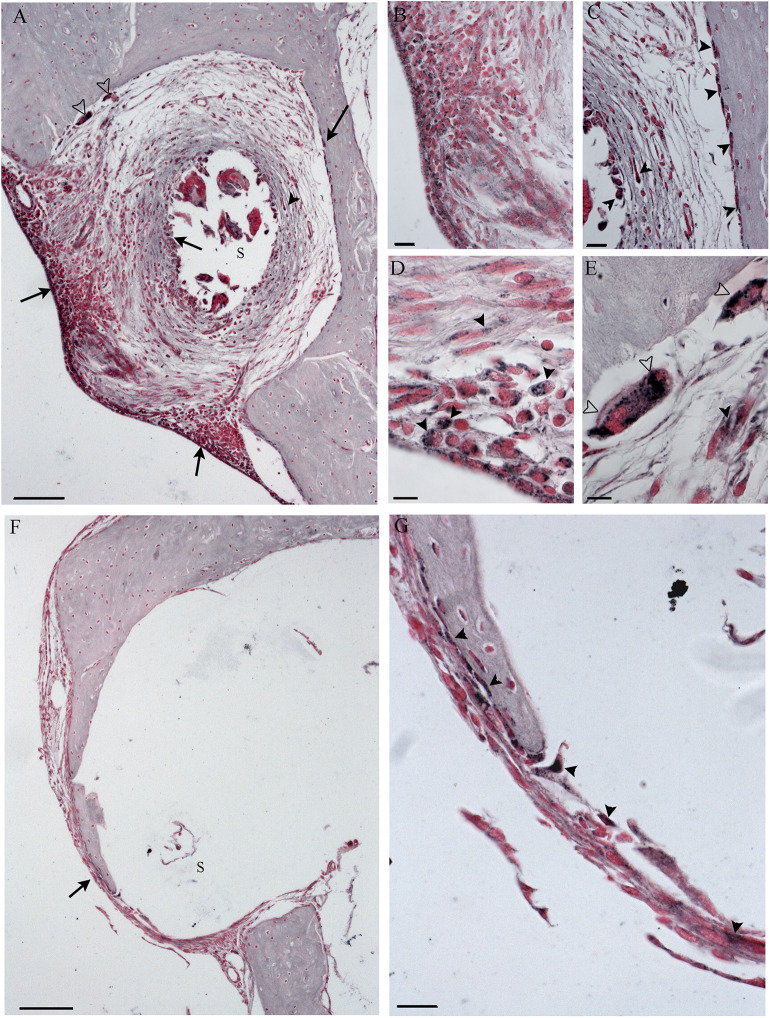
Immunohistochemistry with IL-6 in the cochleostomy site after 60 days, according to treatment. NER group **(A–E)**; DER group **(F,G)**. Arrows indicate staining inside the fibrotic tissue; black arrowheads indicate stained cells; white arrowheads indicate stained FGB. S, rod site; Scale bars, 100 μm **(A,F)**, 50 μm **(B,C,G)**, and 10 μm **(D,E)**.

**Figure 10:**
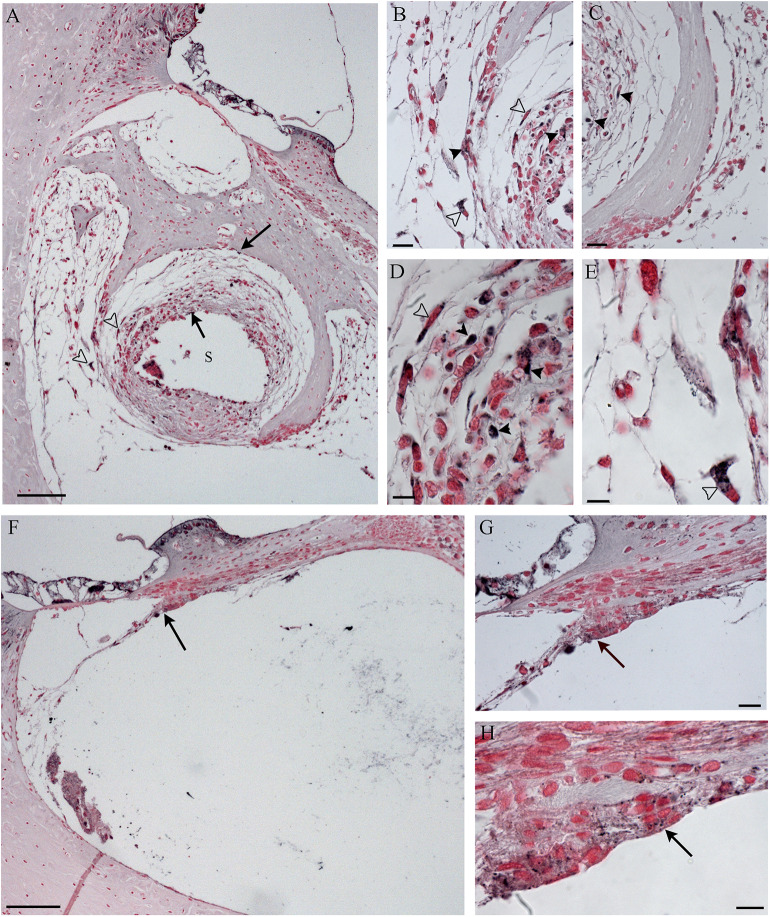
Immunohistochemistry with IL-6 in the scala tympani along the basal turn of the cochlea, according to treatment. NER group **(A–E)**; DER group **(F–H)**. Arrows indicate staining inside the fibrotic tissue; black arrowheads indicate stained cells; white arrowheads indicate stained FGB. S, rod site; Scale bars, 100 μm **(A,F)**, 20 μm **(B,C,G)**, and 10 μm **(D,E,H)**.

No TNFalfa staining was detected in the tissue growth in both groups of treatment (data not shown). In NER, it was possible to recognize immune cells [lymphocytes and foreign body giant cells (FBGCs)] in the inflammatory tissue, mostly composed of fibroblasts and bone, both in scala tympani and around the cochleostomy (Figures 8–10). The FBGCs are mostly found near new bone growth (Figure 8). In the NER group, IL-6 staining was present in all inflammatory tissue growth around the cochleostomy and in the scala tympani, mostly in the fibrotic tissue surrounding the silicon rod (Figures 9A–E and 10A–E), into the FBGCs close to new bone growth (Figure 9E) and in the cells surrounding the marginal cochleostomy area (Figure 9C). In the DER group, the inflammatory reaction was significantly lower (Figures 9F,G and 10F–H).

### Correlation of Hearing Loss in Animal Studies With Histological Findings

The NER group showed a positive correlation between the CAP recorded at all frequencies (at day 0 and day 60) and the amount of tissue detected around the cochleostomy hole [fibrosis with *R*_(6)_ = 0.8286, *p* < 0.05 and new bone with *R*_(6)_ = 0.8986, *p* < 0.05]. Concerning the scala tympani occlusion, the fibrosis was positively correlated only with the CAP higher frequencies recorded at the day 60 [*R*_(6)_ = 0.8117, *p* < 0.05]. In the DER group, a significant correlation between tissue growth in the scala tympani [total tissue with *R*_(7)_ = 0.8929, *p* < 0.05 and fibrosis *R*_(7)_ = 0.8571, *p* < 0.05] and the CAP click was recorded at day 0.

At linear regression, DERs did not impact TS outcomes at post-operative day 60 for the 4–8 kHz frequencies. On the contrary, for the 16–32 kHz frequencies, the positioning of DERs was associated with a hearing recovery of 26.6 dB (95%CI −42, −11 dB; *p* < 0.01). Furthermore, the area of bone formation on the cochleostomy external surface positively impacted TS, while a negative effect on hearing was observed in the case of fibrous tissue formation on the external surface of the cochleostomy and fibrosis area in the scala tympani, at 1,000 μm from the cochleostomy (Table 1).

**Table 1:** Linear regression.

**Variable**	**Coefficient**	**95% CI**	***p*-value**
DER	−26.6	[−42, −11]	<0.01
Bone area (μm^2^), external cochlear surface	−0.0001	[−0.00012, 0.0000093]	0.03
Fibrosis area (μm^2^), external cochlear surface	0.00004	[−0.000069, −0.0000083]	0.02
Fibrosis area (μm^2^), scala tympani, at 1,000 μm	2.24	[0.93, 3.6]	<0.01

*Multiple R^2^, 0.788; F statistic, 7.41 on 4 and 8 DF; p-value, 0.00845*.

## Discussion

### Electrophysiology

The main issue in CI electrode arrays is to design new less traumatic electrodes, able to avoid all side effects leading to CI outcome failures (34). In the DER group, the CI insertion trauma caused about 20 dB SPL of TS at click and at the 4–8 kHz frequencies, and about 35 dB SPL at the 16–32 kHz frequencies. This damage was higher than that detected in previous studies in the same animal model (7); thus, it was suitable for studies of chronic effects of dexamethasone-eluting CI rods. In the course of time, at all frequencies tested, a decrease in TS was detected up to 30 days; there was also an increase at 60 days, although not significant. Similar results were previously reported in the same animal model treated with dexamethasone but with a different surgical approach (8–10). In the NER group, the TS caused by CI insertion trauma was higher than in the DER one; thus, it is possible that DERs have exerted a positive effect in hearing ability preservation.

### Tissue Growth and Immunoreaction

The histological analyses supported the hypothesis that dexametasone protected the cochlea from the inflammatory reaction, because a lower tissue growth was observed in the scala tympani in the DER group in comparison to the NER one. Moreover, only in NER was a significant positive correlation detected between the occlusion of scala tympani and the hearing loss detected at day 60, while in the DER group, a significant positive correlation was detected between the occlusion and the insertion trauma. In a previous study, it was demonstrated that Guinea pigs implanted with eluting silicon electrode arrays (containing from 0.1 to 10% of dexamethasone) showed significantly less fibrosis in the scala tympani and reduced loss of number of synapses and nerve fibers. The ABR and CAP thresholds were parallel. In addition, in Guinea pigs implanted with the array containing 10% of dexamethasone, CAP thresholds were significantly reduced after 90 days post-surgery (8). In humans, it was shown that when used for a long time, CI often causes fibrotic reaction (16) and affects the inner ear tissues, mostly hair cells and dendritic processes (residual hearing) (35).

Concerning the tissue growth, an immunoreaction was observed in both treatment groups. The tissue growth observed after CI insertion may be due to foreign body reaction to the rod, triggered by monocytes and bone fragments carried inside the scala tympani after CI insertion (15, 36). In humans, the histological analyses showed the presence of lymphocytes (B and T), macrophages, and FBGCs in the tissue growth around platinum or silicon particulate, supposedly derived from electrode degeneration (14, 15, 35). In the present study, the infiltration of immune cells was confirmed. In addition, in the NER group, which showed higher immunoreaction, the silicon rod seemed to be replaced with the fibrous tissue, although no silicon particles were detected within the FBGCs. In the DER group, the release of dexamethasone apparently reduced the inflammation.

In the DER group, the new bone formation was significantly lower than in the NER group, especially near the cochleostomy hole, as reported by other authors (10, 14, 16). Development of the extracellular matrix is promoted by fibroblast secretion triggered by macrophages (37): new bone formation is directly related to fibrosis and to the damage extent (35). Our results support the hypothesis that dexamethasone successfully interferes with new bone formation. According to previous studies, dexamethasone is able to inhibit the production of collagen and fibronectin promoting osteoblast apoptosis and autophagy of osteoblast-like cells (38).

In the DER group, the severity of the inflammatory reaction was lower than that in the NER group, with less IL-6 expression and infiltration of immune cells such as FBGCs. The IL-6 is an inflammatory cytokine expressed by several cell types, such as damage-associated molecular patterns (DAMPs), endothelial cells, macrophages, and fibroblasts: its expression is significantly reduced by glucocorticoids (39, 40). The expression of IL-6 detected in the tissue growth around the rod in the NER group may be related to DAMP cells, fibroblasts, or FBGC activity, and the presence of dexamethasone in the DER group prevents their activation and then the recruitment of immune cells. The lower production of new bone in the DER group is then supported by the lower amount of FBGCs that are known to exhibit osteoblast-like activity (41). The formation of FBGC is due to macrophage fusion triggered by chronic inflammation developed around the foreign material (40), supporting the minor side effects in the DER group.

### Correlation of Hearing Loss in Animal Studies With Histological Findings

The significant positive correlation between CAP TSs, recorded as insertion trauma at day 0 and hearing loss at day 60, and tissue growth around the cochleostomy in the NER group may support the use of dexamethasone to reduce these side effects, since a significant lower amount of new bone has been detected in the DER group. Moreover, the significant positive correlation between the hearing loss at day 60 and the scala tympani occlusion in the NER group is apparently prevented by dexamethasone release. However, the significant positive correlation between the tissue growth in the scala tympani after 60 days and the insertion trauma recorded at click supports the hypothesis that this chronic reaction may be avoided by increasing the amount of dexamethasone administered during the surgery. In a previous study, a significant correlation between the tissue growth and the impedance was detected in Guinea pigs implanted with similar DERs for 90 days, but no correlation was detected between the hearing loss and the tissue growth (10). This discrepancy may be due to a different study design, for example, to the method by which the hearing ability was measured (CAP vs. acoustically evoked auditory brainstem response). In our animal model, no significant reduction in neuronal density was detected in both groups, supporting the hypothesis that the addition or not of dexamethasone to CI electrodes had no effect on neurons of the spiral ganglion. Similar results were shown in the same animal model implanted with dexamethasone-eluting electrodes embedded with a range of drug from 1 to 10% (11) and in cat models, in which no relation between the degree of cochlear inflammation and the ganglion cell density was found (42). The neuronal density of NER and DER groups was similar to neuronal density observed in normal Guinea pigs by Wrzeszcz et al. (43), supporting the hypothesis that soft surgery did not damage spiral ganglion neurons. Bas and colleagues demonstrated that electrode insertion trauma causes the loss of synapses and damages of nerve fibers (8), probably because they used a different and invasive surgery approach.

## Conclusions

In humans, fibrotic tissue and new bone formation constantly detected around the cochleostomy hole did not appear to impair CI outcome, except in the case of hybrid implants, and the lower density of neurons did not significantly reduce speech perception (4). Nonetheless, a thicker fibrous tissue within the cochlea may play a role in loss of residual hearing, and the preservation of low frequencies has a pivotal role in improving speech perception (44). In conclusion, the dexamethasone release is a good strategy to counteract the inflammatory reaction, but further studies on mechanisms underlying these side effects is essential to develop improved electrodes based on dexamethasone release that will be able to completely avoid the occurrence of inflammation.

## Data Availability Statement

The datasets generated for this study are available on request to the corresponding author.

## Ethics Statement

The animal study was reviewed and approved by All animal tests were approved according to Italian guidelines provided in DL 116/92, with reference to European Economic Community directive 86-609.All experiments were approved by the Ethics Committee for Animal Usage of the University of Ferrara (Ferrara, Italy), registration no. 9982/2010.

## Author Contributions

ES, AM, and LA contributed to the conception and design of the study. LA and ES performed the electrophysiological and histological analyses. DC, SN, and MA performed the statistical analysis. LA wrote the first draft of the manuscript. MC, GB, FR, EG, and MC wrote sections of the manuscript. All authors contributed to manuscript revision, and read and approved the submitted version.

## Conflict of Interest

The authors declare that this study received funding from MED-EL Hearing Implants (Innsbruck, Austria). The funder was partially involved in the study design, and provided the electrode used in the experiments. The funder was not involved in collection, analysis, interpretation of data, the writing of this article or the decision to submit it for publication. The authors declare that the research was conducted in the absence of any commercial or financial relationships that could be construed as a potential conflict of interest.
